# Plantar pressures and relative metatarsal lengths in older people with and without forefoot pain

**DOI:** 10.1186/1757-1146-5-S1-P16

**Published:** 2012-04-10

**Authors:** Hylton B Menz, Mohammad R Fotoohabadi, Shannon E Munteanu, Gerard V Zammit, Mark F Gilheany

**Affiliations:** 1 Musculoskeletal Research Centre, La Trobe University, Bundoora, Victoria 3086, Australia; 2 Department of Podiatry, La Trobe University, Bundoora, Victoria 3086, Australia

## Background

It has been suggested that plantar forefoot pain (‘metatarsalgia’) may be caused by the presence of abnormally long lesser metatarsals leading to excessive loading of the metatarsal heads when walking. However, evidence to support this proposed mechanism is limited. Therefore, the objective of this study was to determine whether plantar pressures during gait and the relative lengths of the lesser metatarsals differ between older people with and without plantar forefoot pain.

## Materials and methods

**Figure 1:**
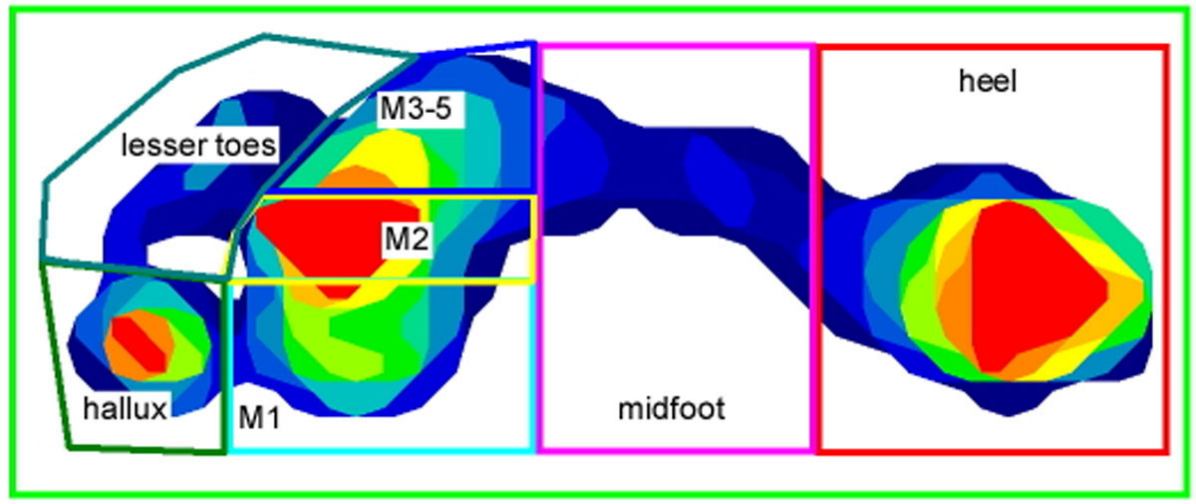
Mask template used for plantar pressure analysis.

Dynamic plantar pressure assessment during walking was undertaken using the Tekscan MatScan® system in 118 community-dwelling older people (44 males and 74 females, mean age 74.0 years, standard deviation [SD] 5.9), 43 (36%) of whom reported current or previous plantar forefoot pain. Seven individual “masks” were constructed to determine peak pressures under the hallux, the lesser toes, metatarsal head 1, metatarsal head 2, metatarsal heads 3 to 5, the midfoot and the heel (see Figure 1). The relative lengths of metatarsals 1 to 5 were determined from weightbearing dorsoplantar x-rays using the Maestro [1] and Coughlin [2] techniques.

## Results

There were no differences between the groups for age, bodyweight or walking speed. Participants with current or previous plantar forefoot pain exhibited significantly greater peak plantar pressure under metatarsal heads 3 to 5 (1.93 [SD 0.41] versus 1.74 [0.48] kg/cm^2^, p=0.032; Cohen’s *d* = 0.42 - medium effect). However, there were no differences in relative metatarsal lengths between the groups.

## Conclusions

Older people with current or previous forefoot pain display greater peak plantar pressures under the lateral metatarsal heads when walking, but do not exhibit relatively longer lesser metatarsals. Other factors may be responsible for the observed pressure increase, such as reduced range of motion of the metatarsophalangeal joints and increased stiffness of plantar soft tissues.
